# Safflower Seed Extract Attenuates the Development of Osteoarthritis by Blocking NF-κB Signaling

**DOI:** 10.3390/ph14030258

**Published:** 2021-03-12

**Authors:** Seong Jae Han, Min Ju Lim, Kwang Min Lee, Eunjeong Oh, Yu Su Shin, Seokho Kim, Joong Sun Kim, Seung Pil Yun, Li-Jung Kang

**Affiliations:** 1 Department of Biomedical Sciences, Ajou University Graduate School of Medicine, Suwon 16499, Korea; hsj2018@ajou.ac.kr (S.J.H.); minju6021@ajou.ac.kr (M.J.L.); eunjeong8836@ajou.ac.kr (E.O.); 2 Department of Pharmacology, Ajou University School of Medicine, Suwon 16499, Korea; 3 Degenerative InterDiseases Research Center, Ajou University School of Medicine, Suwon 16499, Korea; 4 Department of Life Science and Environmental Biochemistry, Pusan National University, Miryang 50463, Korea; leekm@pusan.ac.kr; 5 Department of Medicinal Crop Research, National Institute of Horticultural and Herbal Science, Rural Development Administration, Eumseong 369-873, Korea; totoro69@korea.kr; 6 Department of Health Sciences, The Graduate School of Dong-A University, Busan 49315, Korea; 7 Herbal Medicine Resources Research Center, Korea Institute of Oriental Medicine, Naju-si, Jeollanam-do 58245, Korea; 8 Department of Pharmacology and Convergence Medical Science, Institute of Health Sciences, School of Medicine, Gyeongsang National University, Jinju 52727, Korea

**Keywords:** N-feruloyl serotonin, N-(p-coumaroyl) serotonin, osteoarthritis, nuclear factor-κB

## Abstract

Although safflower seed extract exhibits pharmacological activity against various diseases, the effects of its individual compounds on osteoarthritis (OA) have not been elucidated. Here, we evaluated the effects of these extracts and their single compounds on OA. N-(p-Coumaroyl) serotonin and N-feruloyl serotonin, main components of safflower seed extract, were isolated by high-performance liquid chromatography. Under in vitro OA mimic conditions, the expression of the matrix metalloproteinases (MMPs) MMP3/13 and a disintegrin and metalloproteinase with thrombospondin motifs (ADAMTS) ADAMTS5 were reduced in mouse chondrocytes treated with safflower seed extract. Furthermore, the oral administration of safflower seed extract attenuated cartilage destruction in a mouse OA model induced by destabilization of the medial meniscus. N-(p-Coumaroyl) serotonin and N-feruloyl serotonin, but not serotonin, reduced MMP3, MMP13, and ADAMTS5 expression in IL-1β-treated chondrocytes. Additionally, they significantly blocked the nuclear factor-κB (NF-κB) pathway by inhibiting IκB degradation and p65 phosphorylation. Our results suggest that safflower seed extract and its single compounds can attenuate cartilage destruction by suppressing MMP and ADMATS5 expression. The anti-arthritic effects are mediated by NF-κB signaling and involve the inhibition of IκB degradation and p65 phosphorylation. These results indicate that safflower seed extract may serve as a novel therapeutic agent against OA.

## 1. Introduction

Osteoarthritis (OA) is a common joint disease that can affect one or multiple joints [1]. The pathological features of OA include cartilage degradation, joint stiffness, pain, and swelling. The pathological mechanism of cartilage destruction in OA involves the upregulation of catabolic factors, such as matrix metalloproteinases (MMPs), the primary enzymes that promote cartilage degradation [2]. These catabolic factors are induced by pro-inflammatory cytokines such as IL-1β, which plays a pivotal role in cartilage destruction and is involved in OA progression [3,4,5]. IL-1β has been shown to induce the expression of several catabolic factors, including MMP3, MMP13, and disintegrin and metalloproteinase with thrombospondin motif 5 (ADAMTS5), in mouse chondrocytes and promote cartilage destruction [4].

IL-1β mediates these catabolic activities in chondrocytes by activating nuclear factor (NF)-κB signaling, which subsequently promotes OA development [6]. NF-κB acts as a transcription factor and plays a regulatory role in biological processes such as inflammation, cell proliferation, and cell differentiation [7,8]. Previous studies have reported that the NF-κB signaling pathway is involved in the development of OA via the downstream activation of catabolic factors in primary chondrocytes, including increased expression of MMP3 and MMP13 [9,10].

IκB is a member of the protein complex that inhibits the NF-κB signaling pathway by binding to the NF-κB complex [11,12]. However, IL-1β promotes the degradation of IκB, resulting in the translocation of NF-κB into the nucleus, thus stimulating NF-κB-specific gene expression [13]. In OA, NF-κB activated by IL-1β (via the degradation of IκB) induces the expression of MMP3 and MMP13, promoting OA progression [6,14]. Although several drugs targeting NF-kB signaling have been developed for the treatment of OA, most are associated with serious gastrointestinal adverse effects [15]. Thus, there is a need for safe medicines using natural materials for OA treatment.

Numerous pharmacologically active natural extracts have potential for clinical applications. Studies have shown that *Boswellia serrata*, *Arnica montana*, Apismellifera, *Psoralea corylifolia*, Rhizome coptides, Betulae cortex, harpagophytum, phellodendron amurense, *Symphytum officinalis*, and *Withania somnifera* reduce the expression of proinflammatory cytokine-induced MMPs by inhibiting NF-κB [16,17]. However, the underlying mechanism remains elusive as the observed effects are attributed to mixed compounds. Therefore, a single compound is more useful for mechanism elucidation and drug development than the total active natural extract.

Safflower (*Carthamus tinctorius* L.) is a perennial plant, distributed worldwide, including China, India, Southern Europe, and North America. Safflower seed oil is used as cooking oil; the seed is also used in cosmetics and traditional medicine worldwide. It possesses pharmacological effects such as anti-diabetic, anti-inflammatory, and anti-cancer activities [18,19,20,21,22]. Among various single compounds in safflower seed extract, serotonin, a neurotransmitter, plays an important neurophysiological role in regulating behavior, mood, and memory [23]. Serotonin, a flavonoid compound, is present as N-(p-coumaroyl) serotonin and N-feruloyl serotonin in safflower seeds. N-Feruloyl serotonin reduces the oxidation of low-density lipoproteins associated with the inflammatory processes of atherosclerosis [18], whereas N-(p-coumaroyl) serotonin has been used to prevent and treat bone diseases, cardiovascular diseases, glioma, and obesity [24,25,26]. N-(p-Coumaroyl) serotonin has been demonstrated to reduce oxidation and suppress inflammatory cytokine expression by inhibiting the NF-κB signaling pathway [27]. As described above, N-feruloyl serotonin and N-(p-coumaroyl) serotonin are used to treat several diseases; however, it remains unknown whether they are effective in OA treatment.

This study, to the best of our knowledge, is the first to demonstrate the protective roles of N-feruloyl serotonin and N-(p-coumaroyl) serotonin on the pathogenesis of OA. We showed that N-feruloyl serotonin and N-(p-coumaroyl) serotonin prevented the development of OA by inhibiting the expression of MMP3 and MMP13 induced by IL-1β. These inhibitory effects involve a reduction in NF-κB signaling via the blockage of IκB degradation.

## 2. Results

### 2.1. Effect of Safflower Seed Extract on Chondrocyte Viability

**Figure 1:**
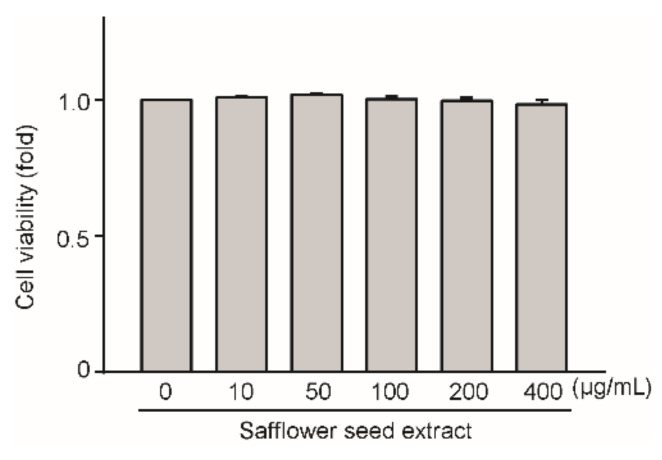
Toxicity of safflower seed extract against chondrocytes. The toxicity of safflower seed extract against chondrocytes was measured using the LDH assay at concentrations of 0, 10, 50, 100, 200, and 400 μg/mL for 24 h.

We determined whether safflower seed extract exerts any toxic effects on chondrocytes by treating the cells with safflower seed extract at concentrations of 10, 50, 100, 200, and 400 μg/mL for 24 h. As shown in Figure 1, the viability of the chondrocytes did not differ from that of the control. The results indicate that safflower seed extract has no toxicity against chondrocytes at the indicated concentrations; thus, all subsequent experiments were performed at concentrations of 100, 200, and 400 μg/mL for 24 h.

### 2.2. Safflower Seed Extract Suppresses IL-1β-Induced Expression of Catabolic Factors in Chondrocytes

**Figure 2:**
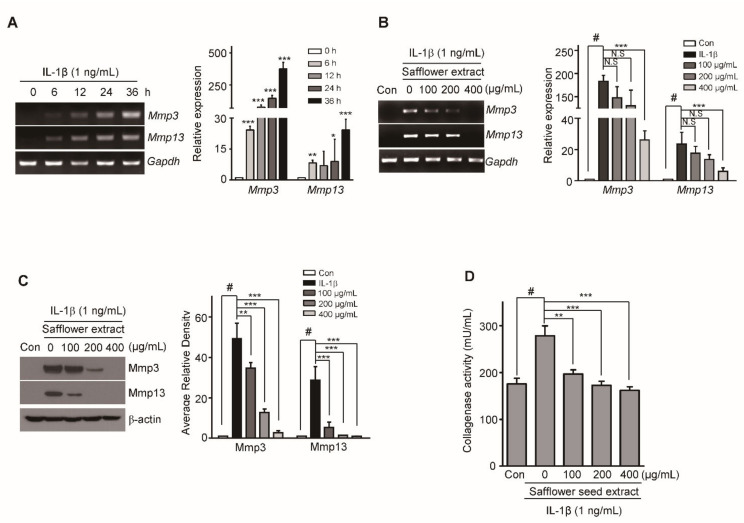
Safflower seed extract reduces matrix metalloproteinases (MMP3 and MMP13) expression in articular chondrocytes induced by IL-1β. Chondrocytes were treated with IL-1β (1 ng/mL) in a time-dependent manner (**A**) and co-treated with different concentrations (100, 200, and 400 µg/mL) of safflower seed extract (**B**). The mRNA expression levels of *Mmp3* and *Mmp13* were measured using RT-PCR (**A**,**B**, left) and qRT-PCR (**A**,**B**, right). The protein expression of MMP3 and MMP13 was determined by western blotting (**C**, left) and quantified by densitometry (**C**, right). *Gapdh* and *β-actin* were used as the loading controls. Collagenase activity in safflower seed extract-treated chondrocytes was evaluated using the Enzchek gelatinase/collagenase assay and detected by ELISA at 482/530 nm (**D**). Data are shown as mean ± SD of each group (*n* = 5). # *p* < 0.05 compared with the control; * *p* < 0.05, ** *p* < 0.01, and *** *p* < 0.001 compared with the IL-1β-treated or safflower seed extract-treated groups.

**Figure 3:**
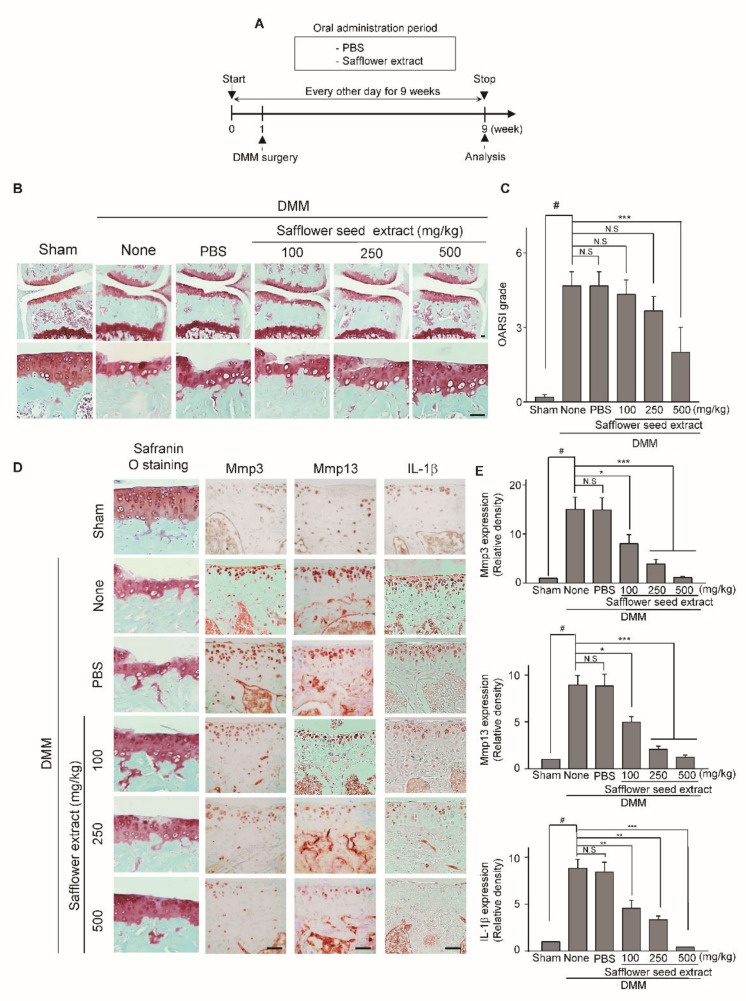
Oral administration of safflower seed extract inhibits cartilage destruction in DMM-induced arthritis mouse model. (**A**) Mice with DMM were orally administered PBS or safflower seed extract three times per week until week nine and analyzed at week nine. (**B**) Analysis of cartilage destruction was performed by safranin O staining. (**C**) Cartilage destruction was determined using the OARSI score at 9 weeks after DMM surgery. The section of cartilage destruction was compared with sham. Scale bars, 100 μm. (**D**) IL-1β, Mmp3, and Mmp13 expression was detected by immunohistochemistry. (**E**) The increased expression of IL-1β, MMP3, and MMP13 in DMM-induced OA cartilage was quantified using ImageJ software v1.60. Data are shown as mean ± SD of each group (*n* = 5). # *p* < 0.05 compared with sham; * *p* < 0.05, ** *p* < 0.01, and *** *p* < 0.001 compared with the group orally administered safflower seed extract.

We previously showed that cartilage destruction is regulated by the expression of catabolic factors such as MMP3 and MMP13 [28,29]. Therefore, we examined whether safflower seed extract reduced the expression of these catabolic factors. The expression of MMP3 and MMP13 increased following IL-1β treatment in a time-dependent manner (Figure 2A). However, when the chondrocytes were co-treated with IL-1β and safflower seed extract for 24 h, the mRNA expression of MMP3 and MMP13 significantly decreased in a dose-dependent manner, as determined using reverse transcription-polymerase chain reaction (RT-PCR) (Figure 2B; left panel) and quantitative RT-PCR (qRT-PCR) (Figure 2B; right panel). A similar decrease was observed when the protein expression of MMP3 and MMP13 was determined by western blotting (Figure 2C; left panel) along with densitometric analysis (Figure 2C; right panel). Moreover, the expression of ADMATS5, a catabolic factor that cleaves aggrecan, was significantly decreased by safflower seed extract (Supplementary Figure S1). Additionally, we evaluated whether safflower seed extract could block IL-1β production and MMP activities. MMP3 and MMP13 possess collagenase activities, and degrade aggrecan, type II collagen, and other extracellular matrix (ECM) components [30]. Increased collagenase activity by IL-1β was significantly reduced by safflower seed extract treatment. Furthermore, we determined the expression of IL-1β, MMP3, and MMP13 by immunohistochemistry. As shown in Figure 3D, the oral administration of safflower seed extract reduced the expression of IL-1β, MMP3, and MMP13 in OA cartilage. These results suggest that safflower seed extract may prevent arthritic progression by reducing the expression of MMP3, MMP13, and ADAMTS5.

### 2.3. Oral Administration of Safflower Seed Extract Suppresses Cartilage Destruction in the DMM-Induced OA Model

To investigate whether the oral administration of safflower seed extract protects against arthritic cartilage degradation in vivo, we evaluated the effect of safflower seed extract in the Destabilization of the medial meniscus (DMM)-induced OA mouse model. Safflower seed extract in Phosphate buffered saline (PBS) (or PBS control) was orally administered to mice three times per week for nine weeks following DMM surgery (Figure 3A). Compared with the PBS control group, the oral administration of safflower seed extract significantly reduced cartilage destruction (Figure 3B). Moreover, the Osteoarthritis Research Association International (OARSI) grades in the group treated with safflower seed extract were significantly lower than those in the PBS control group (Figure 3C). Overall, safflower seed extract provides protection against cartilage destruction observed in the OA mouse model.

### 2.4. HPCL Analysis of Safflower Seed Extract

**Figure 4:**
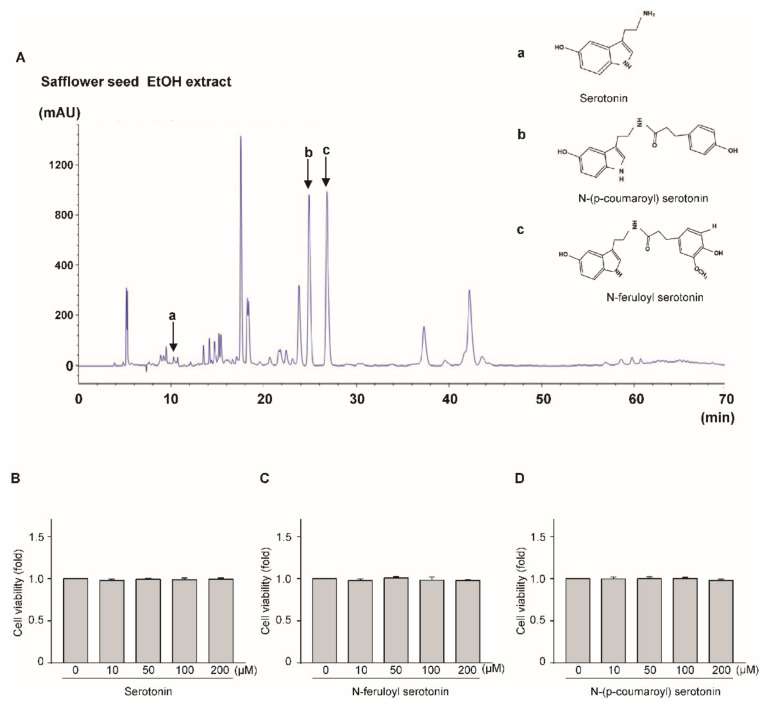
HPLC spectrum of serotonin ^(a)^, N-(p-coumaroyl) serotonin ^(b)^, and N-feruloyl serotonin ^(c)^ in safflower seed extract and standard compounds (**A**). Toxicity of serotonin (**B**), N-feruloyl serotonin (**C**) and N-(p-coumaroyl) serotonin (**D**) against chondrocytes. The toxicity of serotonin, N-(p-coumaroyl) serotonin, and N-feruloyl serotonin against chondrocytes was measured at concentrations of 0, 10, 50, 100, and 200 μM for 24 h using the LDH assay.

Safflower seed extract has been reported to contain large quantities of flavonoid compounds, including serotonin, N-(p-coumaroyl) serotonin, and N-feruloyl serotonin. Here, we quantified serotonin, N-(p-coumaroyl) serotonin, and N-feruloyl serotonin content in safflower seed extract by HPCL (Figure 4A). A comparison with standard compounds revealed that N-(p-coumaroyl) serotonin and N-feruloyl serotonin were the most abundant flavonoids in safflower seed extract (Supplementary Table S2). To determine whether serotonin, N-(p-coumaroyl) serotonin, and N-feruloyl serotonin have toxic effects on chondrocytes, the cells were treated with serotonin at concentrations of 10, 50, 100, and 200 μM for 24 h. As shown Figure 4B–D, serotonin, N-(p-coumaroyl) serotonin, and N-feruloyl serotonin showed no toxicity against chondrocytes at the indicated concentrations. Based on these data, all further experiments were conducted using 100 and 200 μM as treatment concentrations and 24 h as the treatment duration.

### 2.5. N-(p-Coumaroyl) Serotonin and N-Feruloyl Serotonin Suppressed the Expression of Catabolic Factors

**Figure 5:**
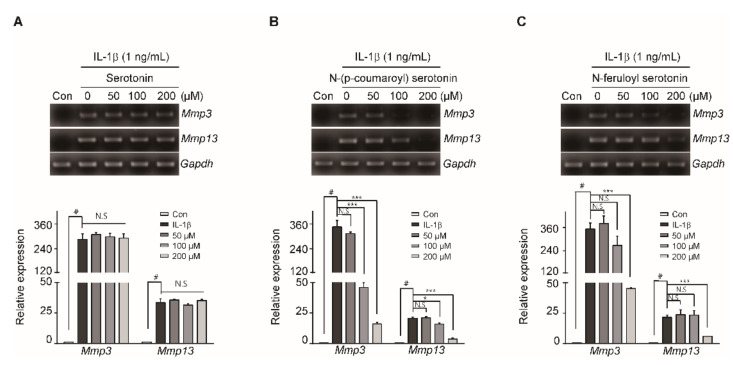
N-(p-coumaroyl) serotonin and N-feruloyl serotonin, but not serotonin, decreased the expression of MMP3 and MMP13 induced by IL-1β in articular chondrocytes. Chondrocytes were treated with various concentrations (0, 50, 100, and 200 µM) of serotonin, N-(p-coumaroyl) serotonin, and N-feruloyl serotonin (**B**). The mRNA expression levels of *Mmp3* and *Mmp13* were measured by RT-PCR (**A**,**B**,**C**; upper panel) and qRT-PCR (**A**,**B**,**C**; lower panel). *Gapdh* was used as the loading control. Data are presented as mean ± SD of each group (*n* = 5). # *p* < 0.05 compared with the control; * *p* < 0.05, *** *p* < 0.001 compared with the IL-1β-, serotonin-, N-(p-coumaroyl) serotonin-, and N-feruloyl serotonin-treated groups**.**

As shown in Figure 2, safflower seed extract reduced the expression of IL-1β-induced MMP3 and MMP13. By HPLC (Figure 4A), serotonin, N-(p-coumaroyl) serotonin, and N-feruloyl serotonin were detected at high levels in safflower seed extract; we determined whether these substances could reduce the levels of catabolic factors, such as MMP3 and MMP13, in chondrocytes in an OA environment using IL-1β. Chondrocytes were treated with various concentrations of serotonin, N-(p-coumaroyl) serotonin, and N-feruloyl serotonin in combination with IL-1β. N-(p-Coumaroyl) serotonin and N-feruloyl serotonin rapidly suppressed the expression of MMP3 and MMP13 as determined by RT-PCR and qRT-PCR (Figure 5B,C), whereas serotonin did not (Figure 5A). This shows that N-(p-coumaroyl) serotonin and N-feruloyl serotonin from safflower seed extract can prevent OA by blocking the expression of MMP3 and MMP13. Moreover, the expression of ADAMTS5 was significantly suppressed by N-(p-coumaroyl) serotonin and N-feruloyl serotonin (Supplementary Figure S2A,B).

### 2.6. Safflower Seed Extract Modulates the NF-KB Signaling Pathway in an In Vitro Model of OA

**Figure 6:**
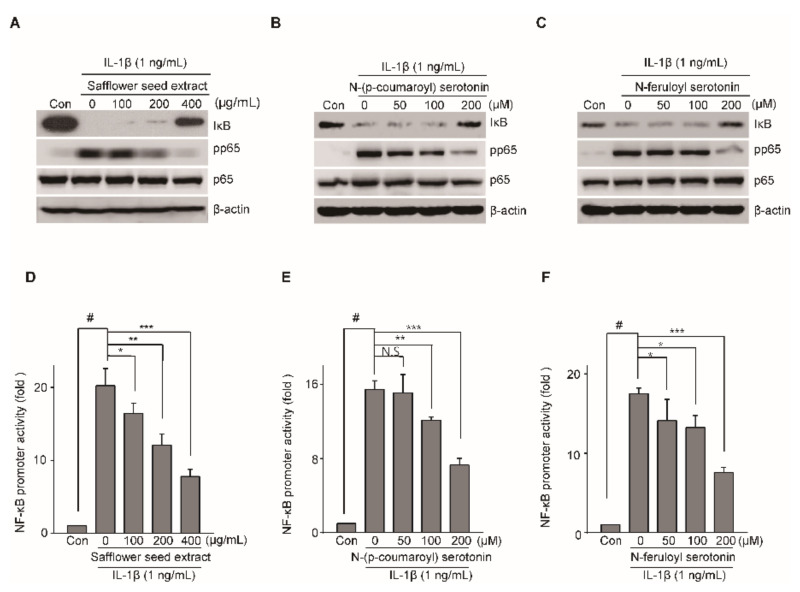
Safflower seed extract, N-(p-coumaroyl) serotonin, and N-feruloyl serotonin regulate IL-1β-induced NF-κB activation. Articular chondrocytes were pretreated with safflower seed extract, N-(p-coumaroyl) serotonin, and N-feruloyl serotonin at various concentrations for 24 h (*n* = 5) before IL-1β (1 ng/mL) treatment for 15 min. Protein expression of IκB and pp65 was determined by western blotting (**A**,**B**,**C**; upper panel) and densitometry (**A**,**B**,**C**; lower panel). β-Actin was used as the loading control. The NF-κB promoter activity was evaluated using the luciferase assay after treatment with safflower seed extract, N-(p-coumaroyl) serotonin, and N-feruloyl serotonin (**D**,**E**,**F**). Data are shown as mean ± SD of each group (*n* = 5). # *p* < 0.05 compared with the control; * *p* < 0.05, ** *p* < 0.01, and *** *p* < 0.001 compared with the IL-1β-treated or safflower seed extract-treated groups.

The IL-1β-mediated NF-κB and MAPK signaling pathway is involved in OA progression [15]; hence, we investigated whether safflower seed extract and serotonin inhibited the NF-κB and MAPK signaling pathway. Articular chondrocytes were pre-incubated for 24 h in the presence or absence of safflower seed extract, N-(p-coumaroyl) serotonin, and N-feruloyl serotonin. These chondrocytes were subsequently treated with IL-1β for 15 min. NF-κB signaling pathway activation was investigated after IκB degradation. Articular chondrocytes were pre-incubated for 24 h in the presence or absence of safflower seed extract, N-(p-coumaroyl) serotonin, and N-feruloyl serotonin. We confirmed that IκB degradation induced by IL-1β was blocked following pre-treatment with safflower seed extract by western blotting (Figure 6A–C) and the subsequent densitometric analysis (Supplementary Figure S4A–C). However, the induced phosphorylation of JNK, ERK, and p38 MAPK by IL-1 had no effect on safflower seed extract treatment of chondrocytes (Supplementary Figure S3). We determined whether NF-κB activation induced by IL-1β was suppressed by treatment with N-(p-coumaroyl) serotonin, N-feruloyl serotonin, and safflower seed extract. The treatment of chondrocytes with IL-1β activated the NF-κB-promoter activity, whereas IL-1β-induced activation of NF-κB promoter was substantially reduced by N-(p-coumaroyl) serotonin (Figure 6E), N-feruloyl serotonin (Figure 6F), and safflower seed extract (Figure 6D). Additionally, the phosphorylation of p65 induced by IL-1β was significantly blocked by N-(p-coumaroyl) serotonin, N-feruloyl serotonin, and safflower seed extract treatment (Figure 6A–C) and the subsequent densitometric analysis (Supplementary Figure S4D–F). Overall, our results suggest that safflower seed extract, N-feruloyl serotonin, and N-(p-coumaroyl) serotonin protect against the pathogenesis of cartilage destruction by blocking IκB degradation, thereby inhibiting the NF-κB signaling pathway.

## 3. Discussion

OA is a degenerative disease characterized by joint stiffness, pain, swelling, and cartilage destruction [30,31]. In OA, damaged cartilage cannot fully recover, and thus the treatment of OA is largely limited to painkillers, which have numerous adverse effects [32,33]. Thus, there is a need to develop safe and effective therapeutic agents for OA. Recently, the treatment of OA using extracts from natural herb compounds has been reported [34].

Safflower seeds contain various flavonoids including serotonin, N-(p-coumaroyl) serotonin, N-feruloyl serotonin, luteolin, and luteolin-7-O-glucoside. However, N-(p-coumaroyl) serotonin and N-feruloyl serotonin are the most abundant flavonoids in safflower seed, and they have been used to treat cancer, cardiovascular diseases, and obesity [21,24,25,35,36,37]. p-Coumaroyl serotonin and N-feruloyl serotonin are used to treat diabetes [36]. 

Polyphenols, two or more hydroxyl groups attached to a phenyl group, have strong antioxidant and anti-inflammation properties, and they are used to treat several diseases [38,39]. Figure 4A shows that serotonin has one hydroxyl group, whereas N-(p-coumaroyl) serotonin and N-feruloyl serotonin have two hydroxyl groups, which improve their pharmacological properties relative to those of serotonin. N-Feruloyl serotonin can reduce the oxidation of low-density lipoproteins associated with the inflammatory process in atherosclerosis [18]. In addition, N-(p-coumaroyl) serotonin has been reported to reduce oxidation and decrease inflammatory cytokine secretion by suppressing the NF-kB signaling pathway [27]. However, the pharmaceutical effects of N-feruloyl serotonin and N-(p-coumaroyl) serotonin against OA have not yet been elucidated. Therefore, here, we performed HPLC and other experiments by targeting N-(p-coumaroyl) serotonin, N-feruloyl serotonin, and serotonin. Safflower seed extract and its single compounds (N-(p-coumaroyl) serotonin, N-feruloyl serotonin, and serotonin) attenuate cartilage degradation by inhibiting the expression of catabolic factors. Furthermore, safflower seed extract suppressed OA development in the DMM-induced OA model. The action mechanism of safflower seed extract and single compounds involves the regulation of NF-κB transcription through the IκB-p65 signaling pathway. 

IL-1β is known to modulate the expression of catabolic factors in mouse chondrocytes [40,41]. Therefore, mouse chondrocytes can be used to mimic experimental OA in vitro. IL-1β treatment of chondrocytes upregulates the expression of catabolic factors, including MMP3 and MMP13 [42,43,44,45]. These proteases act as collagenase and promote the degradation of type II collagen, after cartilage destruction [30]. Clinical data have shown that MMP3 and MMP13 are highly expressed in the cartilage of patients with arthritis, resulting in OA progression [44]. In the present study, safflower seed extract suppressed the expression of MMP3, MMP13, and ADAMTS5 induced by IL-1β stimulation in chondrocytes. Moreover, the oral administration of safflower seed extract attenuated cartilage destruction in a DMM mouse model of OA progression. Additionally, N-(p-coumaroyl) serotonin and N-feruloyl serotonin contained in safflower seed extract suppressed the expression of MMP3 and MMP13 induced by IL-1β in chondrocytes, but not serotonin, in vitro.

NF-κB, a transcription factor, plays an important role in cellular responses to various stimuli such as stress, chemokines, and pro-inflammatory cytokines [46,47]. NF-κB activation is initiated by the degradation of the IκB protein inhibitor bound to NF-κB [48,49]. Following the degradation of IκB by various stimuli, p65, an NF-κB complex, is phosphorylated and translocated into the nucleus where NF-κB-target genes, including MMP3, MMP13, and ADAMTS5 are subsequently upregulated [50,51]. Several studies have reported that the NF-kB signaling pathway promotes the gene expression of catabolic factors that degrade joint cartilage leading to OA progression. Therefore, targeting strategies that block the NF-kB signaling pathway may be a potential option for the treatment of OA. In the present study, the treatment with safflower seed extract, N-(p-coumaroyl) serotonin, and N-feruloyl serotonin blocked IκB degradation and suppressed p65 phosphorylation, and subsequently inhibited NF-κB activation. In conclusion, our study demonstrated that the oral administration of safflower seed extract reduces the progression of OA development by protecting against cartilage degradation. Furthermore, safflower seed extract decreased the expression of catabolic factors (MMP3, MMP13, and ADAMTS5) by blocking IκB degradation and subsequent NF-κB activation. In addition, N-feruloyl serotonin and N-(p-coumaroyl) serotonin suppressed the expression of catabolic factors by inhibiting IκB degradation and subsequent NF-κB activation. Therefore, safflower seed extract, N-feruloyl serotonin, and N-(p-coumaroyl) serotonin may be developed as potential therapeutic agents for OA by regulating the NF-κB signaling pathway.

## 4. Materials and Methods

### 4.1. Reagents and Treatment

We purchased safflower seed extract from Geunyang Oriental Medicine Co. (Sancheong, South Korea). A voucher herbarium specimen has been deposited at the Department of Medicinal Group Research, National Institute of Horticultural and Herbal Science, Rural Development. The samples were identified by the Herbal Crop Research Division. Safflower seeds were dried, ground, and extracted two times using 60 Hz ultrasonic waves in water for 3 h. The solvent was evaporated under vacuum at 40 °C to obtain an extract with a yield of 10.8% (by weight) of the original safflower seeds. The extract was dissolved in dimethyl sulfoxide (DMSO) at 100 mg/mL concentration and in PBS at 200 mg/mL concentration for the in vivo analysis. Serotonin was purchased from Sigma-Aldrich (St. Louis, MO, USA). N-Feruloyl serotonin and N-(p-coumaroyl) serotonin were obtained from Santa Cruz Biotechnology Inc. (Santa Cruz, CA, USA). IL-1β recombinant proteins were purchased from GenScript (Piscataway, NJ, USA). Safflower seed extract, serotonin, N-(p-coumaroyl) serotonin, and N-feruloyl serotonin were dissolved in DMSO, and recombinant proteins were dissolved in sterilized water. Mouse primary articular chondrocytes were treated with IL-1β (1 ng/mL) and co-treated with safflower seed extract at the indicated concentrations (100, 200, and 400 μg/mL) for 24 h.

### 4.2. Primary Culture of Articular Chondrocytes

Articular chondrocytes were obtained from the femoral condyles and tibial plateaus of 5-day-old neonatal mice. All animal experiments were performed with the approval of the Ajou University Animal Care and Use Committee (protocol code 2016-0041). Post-natal day 5 ICR mice were purchased from DBL (Chungbuk, South Korea). Cartilage tissue was digested with 0.2% collagenase type II using a previously described protocol [33]. 

### 4.3. Cytotoxicity Analysis

Before cell treatment, primary cultured chondrocytes were seeded in 96-well plates (9 × 10^3^ cells/well) and incubated for 48 h in Dulbecco’s modified Eagle’s medium (DMEM) with fetal calf serum (FBS). After 24 h, cell viability was assessed using the lactate dehydrogenase (LDH) assay without passage culture. The experiments were conducted using an LDH colorimetric assay kit (BioVision Inc, Milpitas, CA, USA). We used untreated samples (viability of 100%) and Triton X-100-treated samples (viability of 0%) for normalization. The supernatant of the chondrocytes treated with serotonin, N-(p-coumaroyl) serotonin, and N-feruloyl serotonin (10, 50, 100, and 200 µM) for 24 h was analyzed. Viability was calculated using the following formula: 100—(sample LDH-negative control)/(maximum LDH-negative control) × 100. Signals were detected using a SYNERGY H1 microplate reader (Biotek, Winooski, VT, USA) at 495 nm.

### 4.4. RT-PCR and Quantitative RT-PCR Analyses

Total mRNA obtained from primary cultured chondrocytes using TRIzol (Molecular Research Center Inc., Cincinnati, OH, USA) was reverse transcribed to synthesize cDNA for amplification by PCR. The expression of the target genes was then quantified by qRT-PCR using SYBR^®^ premix Ex Taq (TaKaRa Bio, Shiga, Japan). The primer sets used for qRT-PCR are shown in Table S1 (Intron Biotechnology, Gyeonggi-do, South Korea). The expression of each target gene was normalized to that of *Gapdh* and is presented as fold-change relative to the indicated controls.

### 4.5. Western Blotting

Total protein was isolated from primary cultured chondrocytes using RIPA lysis buffer (150 mM NaCl, 1% NP-40, 50 mM Tris pH 8.0, 0.2% sodium dodecyl sulfate, and 5 mM NaF) containing a protease and phosphatase inhibitor cocktail (Roche, Madison, WI, USA). MMP3 and MMP13 were isolated from chondrocyte culture-conditioned medium using trichloroacetic acid, as previously described [33]. The protein extracts were then separated by SDS-PAGE and transferred onto a PVDF membrane by western blotting. The following antibodies were used for western blotting: anti-MMP3 (ab52915; Abcam, Cambridge, UK); mouse anti-MMP13 (ab51072; Abcam); mouse anti-Erk1/2 (610408; Becton Dickinson, Bergen County, NJ, USA); mouse anti-IκB (9242; Cell Signaling Technology (CST),Danvers MA, US); mouse anti-p65 (#6956; CST); mouse anti-pp65 (#13346; CST); mouse anti-p38 (#9212; CST); mouse anti-pp38 (#9215S; CST); mouse anti-c-Jun N-terminal kinase (JNK) (#9252S; CST); mouse anti-pJNK (#9251S; CST), and mouse anti-pErk (#9101S; CST). The protein bands were subsequently detected on the blotted membranes using a secondary antibody and the SuperSignal West Dura kit (Thermo Scientific, Waltham, MA, USA). Erk1/2 was used as a house-keeping protein during the subsequent analysis.

### 4.6. IL-1β Assay and Collagenase Activity Assay

IL-1β was measured using the IL-1β ELISA kit (Koma Biotech, Seoul, South Korea). Primary mice chondrocytes were seeded in 35-mm cell culture plates (2.5 × 10^5^ cell/well) and treated with safflower seed extract and IL-1β for 24 h without passage culture. IL-1β level was quantified in cell lysate according to the manufacturer’s protocol. For collagenase activity assay, the supernatants of primary mouse chondrocyte culture were quantified using the EnzChek Gelatinase/Collagenase Assay Kit (Molecular probes, Carlsbad, CA, USA) after safflower seed extract and IL-1β treatment according to the manufacturer’s protocol. IL-1β and collagenase activities were measured using a Synergy™ HTX Multi-Mode Microplate Reader (BioTeK, Winooski, VT, USA) at OD = 450 nm (IL-1β) and Ex/Em = 480/530 nm (collagenase activity).

### 4.7. Experimental OA Mouse Model and Oral Gavage

Ten-week-old C57BL/6 male mice weighing 18–20 g were housed in temperature-controlled (23 °C) conditions under a 12-h light/ dark cycle. Food and water were regularly provided to the mice. Ten-week-old C57BL/6 male mice were purchased from DBL (Chungbuk, South Korea). C57BL/6 mice were randomly divided into five groups (control, DMM + PBS, DMM + 100 mg/kg, DMM + 200 mg/kg, and DMM + 500 mg/kg). DMM surgery was performed using a standard protocol for the OA mouse model [52,53]. 

### 4.8. Safranin O Staining and Immunohistochemical Analysis

For the subsequent histological analysis, the animals were euthanized by CO_2_ inhalation 10 weeks after the surgery, and mouse knee joints were fixed in 4% paraformaldehyde, decalcified, and embedded in paraffin. Cartilage destruction was followed by safranin O staining and scored according to the OARSI grading system. Immunohistochemistry was performed in mouse knee joint section using anti-MMP3 (ab52915, Abcam), anti-MMP13 (ab51072, Abcam), and anti-IL-1β (CST). Oral administration of safflower seed extract was performed three times a week for 10 weeks, and the mice were sacrificed after the oral gavage.

### 4.9. Luciferase Assay 

The NF-κB and Renilla reporter gene constructs were co-transfected into mouse chondrocytes using LipofectAMINE Plus (Invitrogen, Carlsbad, CA, USA) and treated with IL-1β and co-treated with safflower seed extract, N-feruloyl serotonin, and N-(p-coumaroyl) serotonin as described previously [33]. The transfected cells were incubated for 24 h. Luciferase activity was measured using an assay kit (Promega, Madison, WI, USA) and subsequently normalized with Renilla activity. 

### 4.10. Statistical Analyses

All experiments were performed independently at least three times. Data are presented as mean ± SEM. Statistical significance was measured using a one-way ANOVA with Dunnett’s post-hoc multiple comparison test or a Student’s *t*-test. Statistical difference was evaluated at ^#^ *p* < 0.05 compared with the sham and control groups, * *p* < 0.05, ** *p* < 0.01, and *** *p* < 0.001. Statistical analyses were performed using GraphPad Prism 5 software (GraphPad, San Diego, CA, USA).

## Data Availability

The data presented in this study are available on request from the corresponding author.

## Supplementary Materials

The following are available online at https://www.mdpi.com/1424-8247/14/3/258/s1, Figure S1. Safflower seed extracts inhibited the expression of Adamts5 induced by IL-1β in articular chondrocyte; Figure S2. N-(p-coumaroyl) serotonin and N-feruloyl serotonin suppressed the expression of Adamts5 induced by IL-1β in articular chondrocyte; Figure S3. The effect of safflower seed extract on the MAPK signaling pathway; Figure S4. The effect of Safflower seed extract, N-(p-coumaroyl) serotonin and N-feruloyl serotonin on NF-κB signalling pathway; Table S1. Primer sequence and PCR conditions; Table S2. Content of serotonin (a), N-(p-coumaroyl) serotonin (b) and N-feruloyl serotonin (c) in the ethanol extract of Safflower seed.
