# Supplementary material for: Safflower Seed Extract Attenuates the Development of Osteoarthritis by Blocking NF-κB Signaling
Source: Pharmaceuticals (Basel). 2021 Mar 12;14(3):258. doi: 10.3390/ph14030258 (PMC7999399; doi:10.3390/ph14030258)
Supplement: Supplementary file 1 [file pharmaceuticals-14-00258-s001.zip › Supplementary tables.docx]

**Table S1**. Primer sequences and PCR conditions

| Gene | Origin | Strand | Sequence | Size  (bp) | AT^a^  (°C) |
| --- | --- | --- | --- | --- | --- |
| *Mmp3* | Mouse | ^b^S  ^c^As | 5'-CTGTGTGTGGTTGTGTGCTCATCCTAC-3'  5'-GGCAAATCCGGTGTATAATTCACAATC-3' | 350 | 58 |
| *Mmp13* | Mouse | S  As | 5'-TGATGGACCTTCTGGTCTTCTGGC-3'  5'-CATCCACATGGTTGGGAAGTTCTG-3' | 473 | 58 |
| *Adamts5* | Mouse | S  As | 5′‐TCCTGATGTTGGTGGCTTCAG‐3'  5′‐TGTCTTGGCAAATCCGGTGTA‐3′ | 95 | 58 |
| *Gapdh* | Mouse | S  As | 5'-TCACTGCCACCCAGAAGAC-3'  5'-TGTAGGCCATGAGGTCCAC-3' | 450 | 55 |

^a^AT, annealing temperature; ^b^S, sense; ^c^As, antisense

**Table S2. Content of serotoin (a), *N*-(*p*-coumaroyl) serotonin (b), and *N*-feruloyl serotonin (c) in the ethanol extract of Safflower seed**

| Compound | Content  (mg/kg) | RSD  (%) |
| --- | --- | --- |
| Serotoin (a) | 46.1 ± 2.3 | 8.533 |
| *N*-(*p*-Coumaroyl) serotonin (b) | 415.1 ± 18.9 | 7.881 |
| *N*-Feruloyl serotonin (c) | 606.5 ± 26.9 | 7.668 |
