# Supplementary figures and images for: Safflower Seed Extract Attenuates the Development of Osteoarthritis by Blocking NF-κB Signaling
Source: Pharmaceuticals (Basel). 2021 Mar 12;14(3):258. doi: 10.3390/ph14030258 (PMC7999399; doi:10.3390/ph14030258)

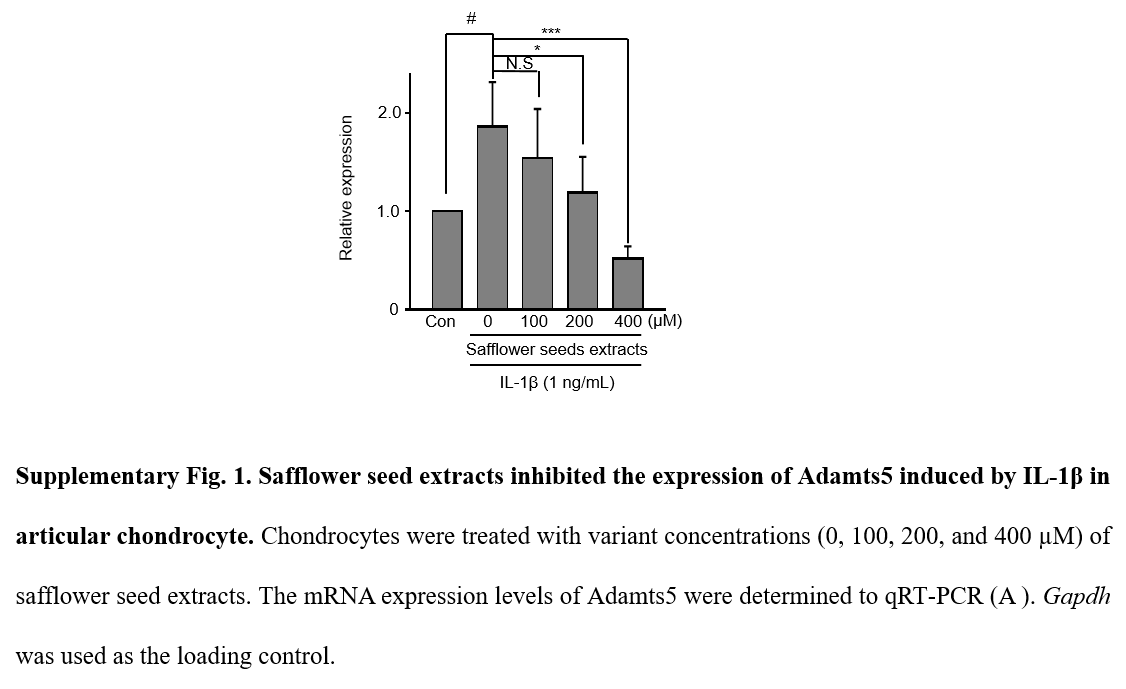

Supplement: Supplementary file 1 [file pharmaceuticals-14-00258-s001.zip › Supplementary figure 1.tif]

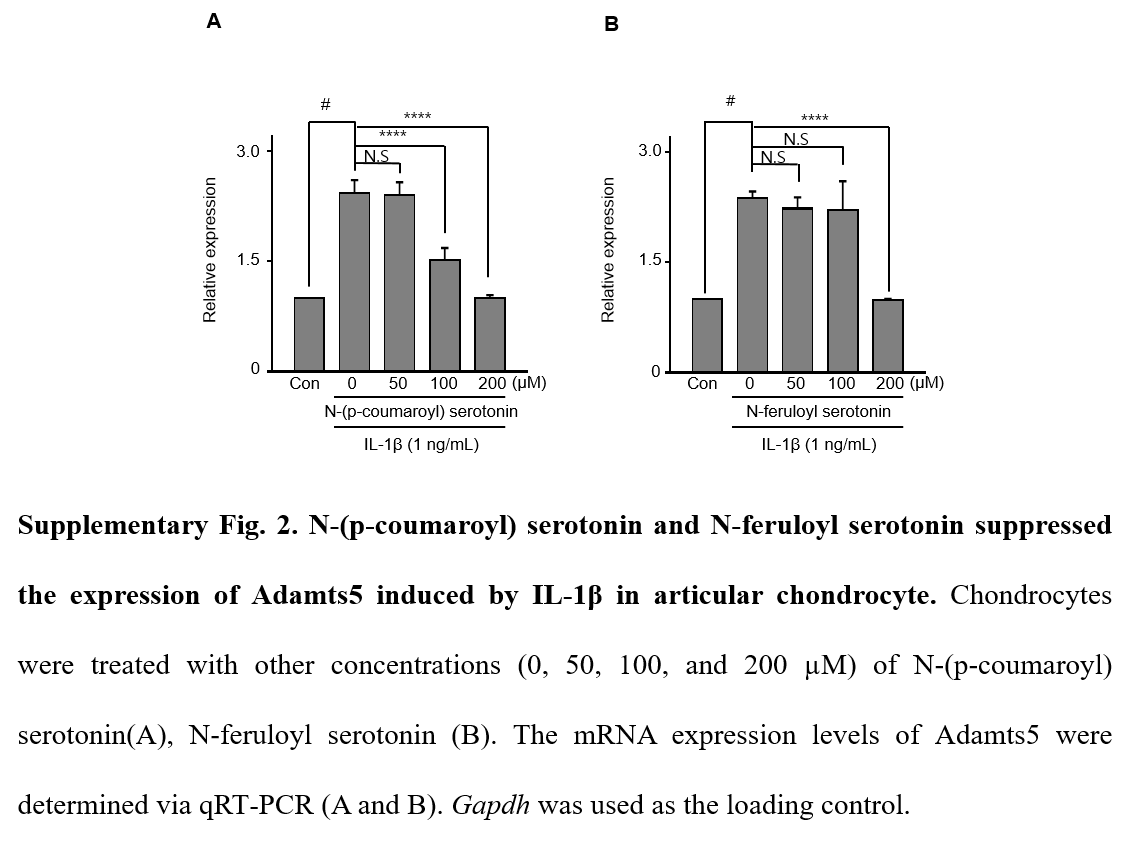

Supplement: Supplementary file 1 [file pharmaceuticals-14-00258-s001.zip › Supplementary figure 2.tif]

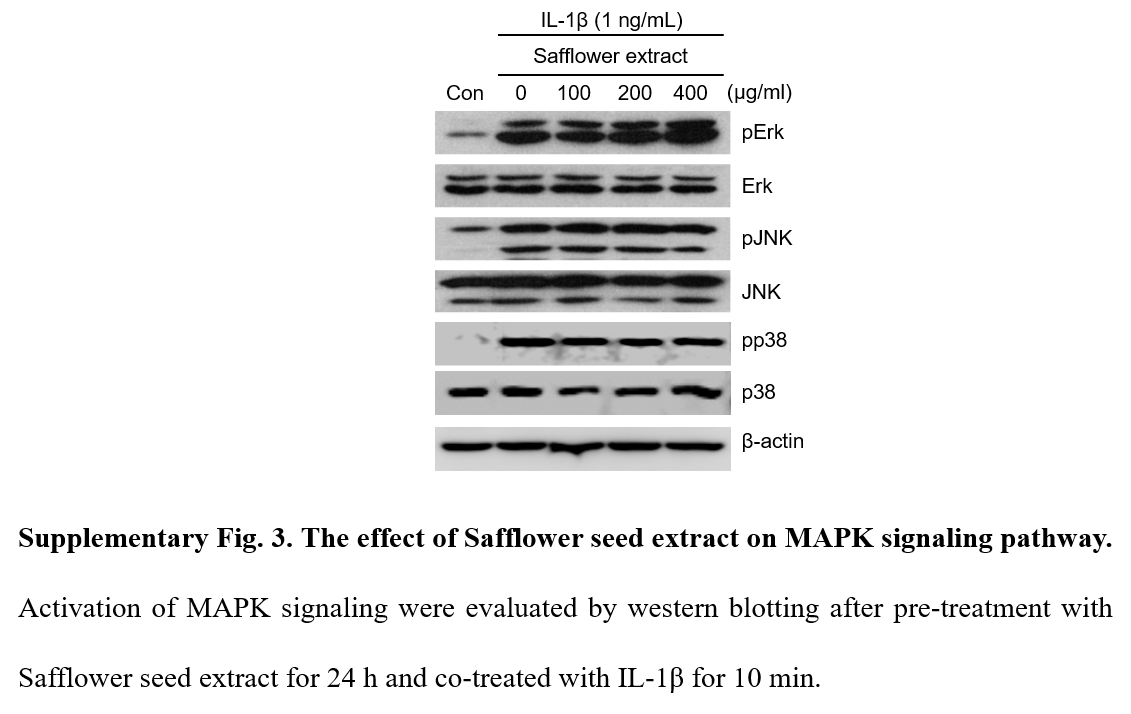

Supplement: Supplementary file 1 [file pharmaceuticals-14-00258-s001.zip › Supplementary figure 3.tif]

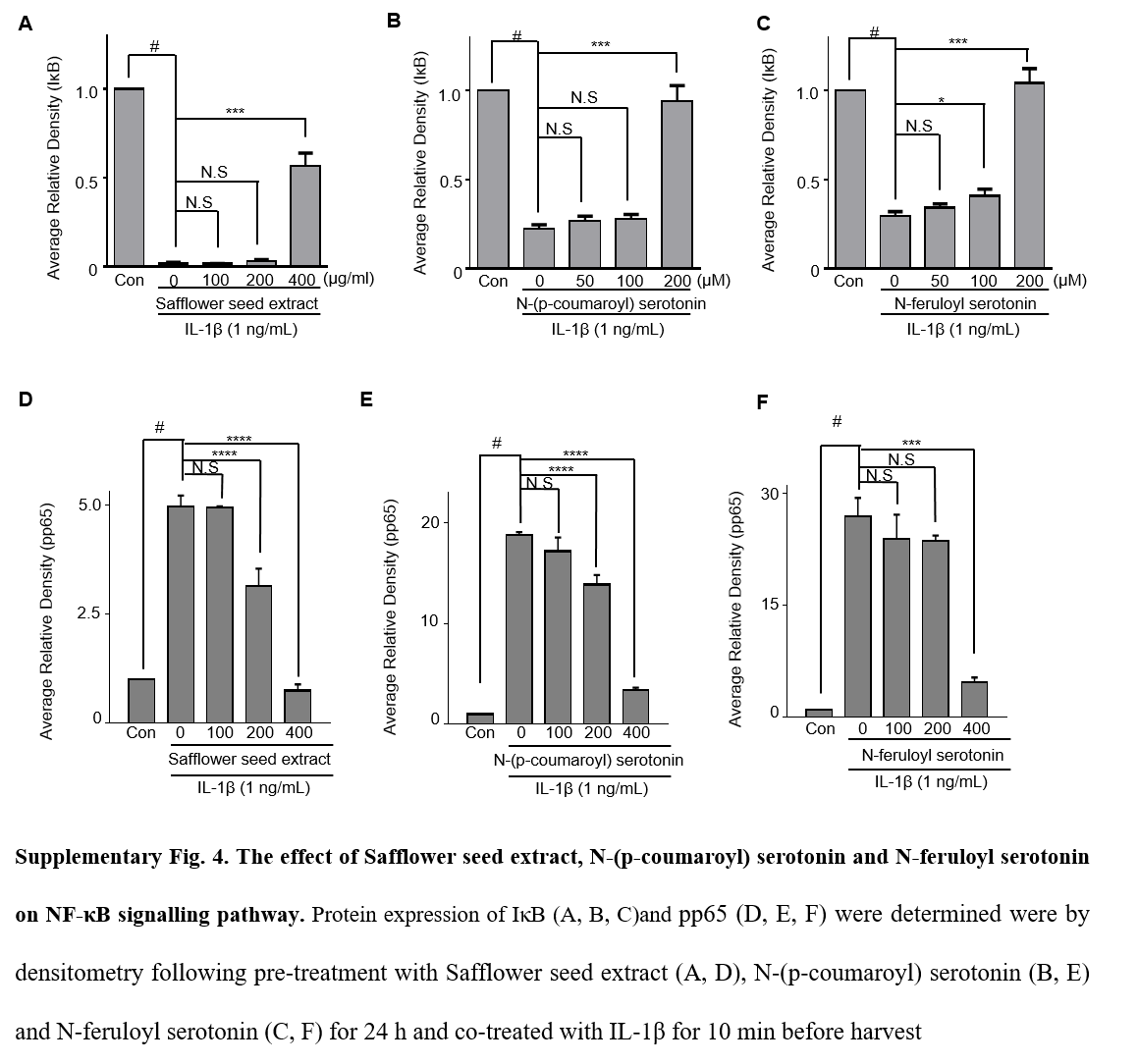

Supplement: Supplementary file 1 [file pharmaceuticals-14-00258-s001.zip › Supplementary figure 4.tif]
